# Developing regional weight-for-age growth references for malaria-endemic countries to optimize age-based dosing of antimalarials

**DOI:** 10.2471/BLT.14.139113

**Published:** 2014-11-20

**Authors:** Daniel J Hayes, Stef van Buuren, Feiko O ter Kuile, D Mikis Stasinopoulos, Robert A Rigby, Dianne J Terlouw

**Affiliations:** a Department of Clinical Sciences, Liverpool School of Tropical Medicine, Pembroke Place, Liverpool, L3 5QA, England.; b Netherlands Organisation for Applied Scientific Research, Leiden, Netherlands.; c STORM Research Centre, London Metropolitan University, London, England.

## Abstract

**Objective:**

To derive regional weight-for-age growth references to help optimize age-based dosing of antimalarials in Africa, the Americas, South-East Asia and the Western Pacific.

**Methods:**

A weight-for-age database was constructed from pre-existing population-based anthropometric data obtained from household surveys and research groups. It contained data collected between 1995 and 2012 on 1 263 119 individuals (909 368 female, 353 751 male) older than 14 days and younger than 50 years in 64 malaria-endemic countries. Regional growth references were generated using a generalized additive model for location, scale and shape by combining data with varying distributions from a range of sources. Countries were weighted by their population at risk of malaria to enable references to be used in optimizing the dosing of antimalarials.

**Findings:**

Large differences in weight-for-age distributions existed between the regions and between the regions and global growth standards. For example, the average adult male from the Americas weighed 68.1 kg – 6.0 kg more than males in South-East Asia and the Western Pacific (average: 62.1 kg). For adult women, the difference was over 10.4 kg: the average was 60.4 kg in the Americas and 50.0 kg in South-East Asia and the Western Pacific.

**Conclusion:**

There were substantial variations in weight-for-age growth curves between malaria-endemic areas. The growth reference charts derived here can be used to guide the evidence-based optimization of aged-based dosing regimens for antimalarials and other drugs often prescribed by age.

## Résumé

**Objectif:**

Obtenir les références de croissance pour le rapport poids/âge pour aider à optimiser la posologie des médicaments antipaludiques en fonction de l'âge dans l'Afrique, les Amériques, le Pacifique occidental et l'Asie du Sud-Est.

**Méthodes:**

Une base de données des rapports poids/âge a été créée à partir des données anthropométriques basées sur les populations et préexistantes, qui ont été obtenues à partir d'enquêtes sur les ménages et de groupes de recherche. Elle contenait des données collectées entre 1995 et 2012 auprès de 1 263 119 personnes (909 368 femmes, 353 751 hommes) âgées de plus de 14 jours et de moins de 50 ans dans 64 pays où le paludisme est endémique. Les références régionales de croissance ont été générées en utilisant le modèle additif généralisé pour la localisation, la taille et la forme en combinant les données avec des distributions différentes à partir de sources diverses. Les pays ont été pondérés en fonction de leur population exposée au paludisme afin que les références puissent être utilisées pour optimiser la posologie des médicaments antipaludiques.

**Résultats:**

Il existait de grandes différences dans les distributions des rapports poids/âge entre les régions et entre les régions et les normes mondiales de croissance. Par exemple, l'homme adulte moyen de les Amériques pesait 68,1 kg – soit 6.0 kg de plus que les hommes vivant dans le Pacifique occidental et l'Asie du Sud-Est (moyenne: 62,1 kg). Pour les femmes adultes, la différence dépassait les 10.4 kg: la moyenne était de 60,4 kg dans les Amériques et de 50,0 kg dans le Pacifique occidental et l'Asie du Sud-Est.

**Conclusion:**

Il existait des variations substantielles dans les courbes de croissance pour le rapport poids/âge entre les zones où le paludisme est endémique. Les tableaux de référence de croissance qui sont obtenus ici peuvent être utilisés pour guider l'optimisation factuelle des schémas posologiques des médicaments antipaludiques en fonction de l'âge et des autres médicaments qui sont souvent prescrits par âge.

## Resumen

**Objetivo:**

Obtener referencias regionales del crecimiento ponderal correspondiente a cada edad para ayudar a optimizar la dosificación basada en la edad de los medicamentos contra la malaria en África, las Américas, el Pacífico Occidental y Asia Sudoriental.

**Métodos:**

Se elaboró una base de datos de pesos correspondientes a cada edad a partir de los datos antropométricos preexistentes basados en la población, obtenidos de encuestas a hogares y grupos de investigación. Esta contenía los datos recogidos entre 1995 y 2012 de 1 263 119 personas (909 368 mujeres y 353 751 varones) mayores de 14 días y menores de 50 años en 64 países donde la malaria es endémica. Se generaron referencias regionales de crecimiento por medio de un modelo aditivo generalizado para la ubicación, la escala y la forma mediante la combinación de datos con diferentes distribuciones procedentes de fuentes diversas. Los países se ponderaron por su población en riesgo de malaria para permitir que las referencias se utilicen en la optimización de la dosificación de los medicamentos contra la malaria.

**Resultados:**

Existían grandes diferencias en las distribuciones del peso correspondiente a cada edad entre las regiones, así como entre las regiones y los estándares de crecimiento mundial. Por ejemplo, el varón adulto promedio de las Américas pesaba 68,1 kg, 6.0 kg más que los varones del Pacífico Occidental y Asia Sudoriental (promedio: 62,1 kg). En las mujeres adultas, la diferencia fue de más de 10.4 kg: el promedio fue de 60,4 kg en las Américas y 50,0 kg en el Pacífico Occidental y Asia Sudoriental.

**Conclusión:**

Hubo variaciones sustanciales en las curvas de crecimiento ponderal correspondiente a cada edad entre las zonas donde la malaria es endémica. Las tablas de referencia de crecimiento aquí obtenidas se pueden utilizar para guiar la optimización con base empírica de los regímenes de dosificación basados en la edad para los medicamentos contra la malaria y otros medicamentos que se prescriben con frecuencia en función de la edad.

## ملخص

**الغرض:**

التوصل إلى معايير مرجعية لنمو الوزن حسب العمر على الصعيد الإقليمي للمساعدة في تحسين جرعات الأدوية المضادة للملاريا حسب العمر في *أفريقيا * والأمريكيتين وجنوب شرق آسيا وغرب المحيط الهادئ.

**الطريقة:**

تم إنشاء قاعدة بيانات معنية بالوزن حسب العمر من بيانات القياسات البشرية السكانية السابقة التي تم الحصول عليها من الدراسات الاستقصائية للأسر المعيشية والفرق البحثية. واحتوت على بيانات تم جمعها في الفترة من عام 1995 إلى عام 2012 حول 1263119 فرداً (909368 أنثى و353751 ذكراً) أعمارهم أكبر من 14 يوماً وأقل من 50 عاماً في البلدان الأربع والستين التي يتوطنها الملاريا. وتم التوصل إلى المعايير المرجعية للنمو على الصعيد الإقليمي باستخدام نموذج مضاف عام للموقع والنطاق والشكل عن طريق دمج البيانات ذات التوزيعات المتباينة من مجموعة من المصادر. وتم ترجيح البلدان حسب سكانها المعرضين للإصابة بالملاريا بغية تمكين استخدام المعايير المرجعية في تحقيق أفضل استفادة من جرعات الأدوية المضادة للملاريا.

**النتائج:**

تبين وجود فروق كبيرة في توزيعات الوزن حسب العمر بين الأقاليم وبين الأقاليم ومعايير النمو العالمية. على سبيل المثال، كان وزن متوسط الذكر البالغ من الأمريكيتين 68.1 كلغم - بزيادة قدرها 6.0 كلغم عن الذكور في جنوب شرق آسيا وغرب المحيط الهادئ (المتوسط: 62.1 كلغم). وبالنسبة للنساء البالغات، كان الفرق أعلى من 10.4 كلغم: كان المتوسط 60.4 كلغم في الأمريكيتين و50.0 كلغم في جنوب شرق آسيا وغرب المحيط الهادئ.

**الاستنتاج:**

تبين وجود تفاوتات كبيرة في منحنيات نمو الوزن حسب العمر بين المناطق التي يتوطنها الملاريا. ويمكن استخدام المنحنيات المرجعية للنمو التي تم التوصل إليها هنا لتوجيه التحسين المستند على البيّنات لنظم الجرعات المستندة على العمر للأدوية المضادة للملاريا وغيرها من الأدوية التي توصف عادة حسب العمر.

## Abstract (zh)

**目的:**

通过使用现有的人体测量数据获得地区年龄别体重的生长参考，以优化在非洲地区、美洲区域、西太平洋地区和东南亚地区基于年龄的抗疟药物剂量。

**方法:**

年龄别体重数据库由基于人群的已有人体测量数据构建，这些数据来自于入户调查和研究团体。它包含了在1995年到2012年间在64个疟疾流行国家收集的大于14天且小于50岁的1263119位个人（909368名女性，353751名男性）的数据。通过将数据与一系列来源范围的各种分布相结合，使用位置、规模和形态的广义相加模型生成地区生长参考。国家按其疟疾高危人群进行加权，使其可以在优化抗疟药物剂量中使用参考。

**结果:**

在区域之间以及区域和全球生长标准之间存在年龄别体重的分布的重大差别。例如，美洲普通成年男性重达68.1公斤，比东南亚和西太平洋男性重6.0公斤（平均：62.1公斤）。成年女性的差别则超过10.4公斤：美洲的平均为60.4公斤，东南亚和西太平洋的平均为50.0公斤。

**结论:**

疟疾流行地区之间年龄别体重生长曲线存在明显的差异。从其中派生的生长参考图表可以用来指导对基于证据的抗疟药物基于年龄的给药方案和通常按年龄配给的药物的给药方案的优化。

## Резюме

**Цель:**

Определить региональные справочные показатели соотношения веса и возраста****для оптимизации возрастных дозировок противомалярийных препаратов в Африке, Америке, Юго-Восточной Азии и Западной части Тихого океана.

**Методы:**

База данных по соотношению веса и возраста была создана на основе уже существующих популяционных антропометрических данных, полученных из исследований домашних хозяйств и от исследовательских групп. В ней содержались данные, собранные в 1995-2012 гг., по 1 263 119 физическим лицам (909 368 женщинам и 353 751 мужчине) в возрасте от 14 дней до 50 лет, проживавших в 64 эндемичных по малярии странах. Региональные справочные показатели роста были получены с использованием обобщенной аддитивной модели с учетом местоположения, масштаба и формы, а также посредством комбинирования данных, полученных по различным распределениям и из различных источников. Полученные показатели для стран были взвешены относительно доли их населения, подверженного риску малярии, с целью получения справочных показателей, подлежащих использованию при оптимизации дозировки противомалярийных препаратов.

**Результаты:**

Были выявлены большие различия в распределении показателей веса и роста как между исследуемыми регионами, так и между данными регионами и мировыми стандартами роста. Например, вес среднестатистического взрослого мужчины в Америкe составлял 68,1 кг — что на 6,0 кг больше веса мужчин в Юго-Восточной Азии и Западной части Тихого океана (в среднем 62,1 кг). Для взрослых женщин эта разница составляла более 10,4 кг: средний вес женщин равнялся 60,4 кг в Америкe и 50,0 кг в Юго-Восточной Азии и Западной части Тихого.

**Вывод:**

Были обнаружены существенные различия в кривых роста соотношения веса к возрасту между эндемичными по малярии районами. Полученные в ходе исследования таблицы справочных показателей роста могут быть использованы в качестве ориентировочных при проведении оптимизации на основе фактических данных схем дозирования противомалярийных и других лекарственных средств, часто назначаемых по возрасту.

## Introduction

Manufacturers of antimalarials recommend that dosing should be based on body weight but, in many low- and middle-income countries, the dose is frequently based on age, which is used as a proxy for body weight because these drugs are often sold over the counter or prescribed in settings without weighing facilities. Unfortunately, the effectiveness and safety of this approach has rarely been assessed and it has been shown that the use of inadequate, age-based dosing regimens is responsible for a considerable proportion of treatment failures.^1^^,^^2^ Since age-based dosing results in much more variability in drug intake than weight-based dosing, over- and underdosing inevitably occur. Substantial improvements could be made by optimizing age-based dosing regimens but this idea has received little attention from manufacturers or policy-makers. Accurate knowledge of the weight-for-age distribution of the population at risk of malaria is vital for establishing the optimal dosing regimen.

Previously, the optimal age-based dosing regimen and drug ratio of a fixed-dose combination of the antimalarials artesunate and amodiaquine for use in sub-Saharan Africa were predicted using weight-for-age data from 88 054 individuals in several African countries.^3^ A recent analysis of the efficacy of artesunate and amodiaquine using pooled data on 5410 patients from 24 studies showed that administered doses of the fixed combination were significantly better with both weight- and age-based regimens than when non-fixed combinations were used. The fixed combination also showed a lower risk of recrudescence.^4^

Currently weight-for-age reference data are lacking for most middle- and low-income countries. The existing global growth standards are the World Health Organization (WHO) Child Growth Standards for children aged 0 to 59 months^5^ – based on the Multicentre Growth Reference Study^6^ – and the 2007 WHO growth reference for school-aged children and adolescents aged 5 to 19 years.^7^ Optimal growth can be assessed against these standards but they are inadequate for establishing optimal dosing of antimalarials as they do not describe how children actually grow at a particular time and place. Since growth varies between regions, regional or country-specific reference data would enable dosing regimens to be tailored to the population affected.

Over the past two decades, a wealth of population-representative anthropometric data has become publicly available from low- and middle-income countries. These data are largely based on national household surveys that monitor standard socioeconomic and health indicators for children younger than 5 years and women of reproductive age.^8^^,^^9^ Research surveys and monitoring and evaluation activities can also provide data on locally representative samples of school-age children, adolescents and adult males. Conventional growth curve modelling methods, such as the LMS method,^10^ and the more recent generalized additive model for location, scale and shape^11^ assume a single data source and distribution. We developed an extension of the generalized additive model using heterogeneous data from representative population samples that reflect different underlying population growth distributions.^12^

Here we present the first application of this extended generalized additive model. We compiled individual-level, weight-for-age data from population-representative data sources in countries where malaria is endemic, principally in three areas: (i) the WHO African Region; (ii) the WHO Region of the Americas; and (iii) the WHO South-East Asia and Western Pacific Regions combined. In addition, malaria-endemic areas in the WHO Eastern Mediterranean Region were initially considered but insufficient data were available. We constructed weight-for-age growth references for the three regions that could be used to optimize dosing regimens for antimalarials and other drugs prescribed by age.

## Methods

We compiled databases of individual-level, anthropometric data from publicly available sources collected between 1995 and 2012 on individuals of both sexes and all ages living in malaria-endemic countries. We also contacted researchers to obtain data on age groups underrepresented in publicly available sources, particularly school-aged children aged 5–15 years and adult males aged 16–50 years.

Malaria-endemic countries were defined according to the *World malaria report 2011*.^13^ We included only those contained in the publication by Hay et al.^14^ Eligible data sets had to contain individual-level data; include data on the country, the year of sampling and individuals’ age, sex and body weight; and have been obtained using random sampling strategies that ensured samples were potentially representative of the age and sex group of interest in the population. Studies of subgroups selected according to nutritional, economic or health criteria were excluded.

We used four principal data sources for anthropometric survey data: (i) Demographic and Health Surveys (DHS); (ii) United Nations Children’s Fund Multiple Indicator Cluster Surveys; (iii) data on adults in low- and middle-income countries that were collected through multistage, cluster-randomized, household surveys using the WHO STEPwise approach to the surveillance of risk factors for major chronic diseases; and, to address gaps in specific countries; and (iv) trial and survey data on population-representative samples from individual studies. Both DHS and Multiple Indicator Cluster Surveys used multistage, random sampling to obtain nationally representative samples and collected data on anthropometric measurements in children aged 0–59 months and in women of childbearing age (i.e. 15–49 years).

We collected information on the data source; the country where data were collected; and each individual’s sex; age, to the nearest day, month or year; and body weight, to the nearest hundred grams, where available. Records with missing observations or obvious errors (e.g. weight under 0 kg or age under 0 months) were excluded. In studies where age was documented in full years, half a year was added to average ages over the year. Data from eligible data sets were merged to create crude weight-for-age databases for Africa, the Americas, and South-East Asia and the Western Pacific. Individuals with weight-for-age *Z* scores that appeared improbable when compared with recent Centers for Disease Control and Prevention (CDC) growth charts were identified.^15^ As the observed spread in weight-for-age *Z* scores between countries and studies was large, the definition of an improbable value was different for different regions: it was: less than −11 standard deviations (SD) or more than +8 SD in Africa, less than −7 SD or more than +5 SD in the Americas, less than−10 SD or more than +5 SD in South-East Asia and the Western Pacific, and less than −6 SD or more than +4 SD in the Eastern Mediterranean. These cut-off values were conservative and reflected biological differences in growth between regions and the fact that median weights in our regions were considerably lower than those in the CDC reference set, which was based on a population from the United States of America. As a final step, only individuals older than 14 days and younger than 50 years were included: the lower limit was set to avoid modelling postpartum weight loss in the first 2 weeks of life and the upper limit, because data from older age groups were scarce.

### Modelling strategy

Details of our modelling method have been published previously.^12^ In brief, country-specific, weight-for-age reference curves were derived from individual-level, population-representative data using a generalized additive model for location, scale and shape. Subsequently, country curves were combined to create regional, weight-for-age, reference curves. To increase the applicability of these regional reference curves for optimizing antimalarial doses, individual country curves were weighted according to the size of the population at risk of malaria caused by *Plasmodium falciparum* or *P. vivax*. Our model (GAMLSS package version 4.2.6 for the statistical package R version 2.15.2, London Metropolitan University, London, United Kingdom of Great Britain and Northern Ireland), which is a generalization of the LMS model, allowed the mean and spread of the weight-for-age distribution to vary between countries. We were able to generate growth charts for the full age range by extrapolating information from neighbouring countries with comparable growth curves, even for countries where information on certain age–sex categories was lacking.

Box 1 provides an overview of the main steps in our modelling strategy. The full data set was split into two parts: 70% for model building and 30% for validation. Subsequently, country- and sex-specific, smoothed, weight-for-age curves were generated. The final models were selected by determining the optimal number of degrees of freedom for the regional curves. Different model distributions were compared to establish which provided the best fit and we examined whether the addition of spline functions would improve the fit. We then used regional mean square error graphs to assess the deviation between various modelled country combinations and global values to determine whether country-level, weight-for-age distributions in each region were sufficiently homogeneous to allow joint modelling of the countries or whether countries needed to be split into groups with similar patterns to model their distributions separately before merging them. The countries in Africa and countries in South-East Asia and the Western Pacific were split into two country groups each, whereas countries in the Americas were modelled together (Appendix A, available from: http://archive.lstmed.ac.uk/4566/). Thereafter, country curves for each region were merged into a single regional weight-for-age reference curve using finite mixture distributions weighted for the size of each country’s population at risk of malaria.^14^ These malaria-specific, weighted, regional references are presented here. The previously published reference for South-East Asia and the Western Pacific was remodelled for individuals older than 14 days and younger than 50 years using updated data sources.^12^

Box 1 **Modelling strategy for generating weight-for-age reference curves for malaria-endemic regions**

- Clean and prepare individual data sets for joint modelling.
- Split the full data set into two parts: 70% for model building and 30% for validation.
- Set up separate databases for each sex.
- Calculate weight-for-age *Z* scores for all individuals and exclude improbable values.
- Establish country modelling groups by examining the divergence of the weight-for-age distributions of individual countries using regional mean square error graphs – countries in each of the following regions were divided into two modelling groups each: Africa, and South-East Asia and the Western Pacific.
- Generate weight-for-age curves for each country.
- Identify the type of weight-for-age distribution.
- Determine whether splines improve the fit of the models.
- Identify the optimal number of degrees of freedom.
- Apply the Schwarz Bayesian criterion as a numerical guide to determine the optimal fit.
- Select final model parameters by comparing country-level worm plots and regional mean square error graphs over a range of numbers of degrees of freedom for all variables.^16^
- Validate the country models by using the validation database to calculate the percentage of individuals in each country with weight-for-age values that corresponded to specific modelled centiles.
- Weight countries according to the population at risk of malaria using Malaria Atlas Project data.^17^
- Calculate weight-for-age reference curves for the three regions by combining country curves using a finite mixture distribution.

WHO: World Health Organization.

A population-at-risk, weighted, average distribution for the three regions together was produced by combining the three regional weight-for-age reference curves, weighted for the population at risk of malaria in each region – including the population at risk in malaria-endemic countries not represented in the modelling database.^14^ This pooled, global, weight-for-age, reference for malaria-endemic regions served as a comparison with other weight-for-age reference curves.

## Results

An overview of the data sets selected for modelling is shown in Table 1. We considered data on 2 275 321individuals from 218 data sources and 77 countries. After limiting data to that collected between 1995 and 2012 and excluding data with missing or invalid entries for country, sex, body weight or age, duplicate data and data from countries for which information on the risk of malaria was missing, the database comprised data on 1 331 936 individuals from 207 sources and 71 countries – 41.5% of the original data were excluded. Improbable weight-for-age *Z* scores were identified, leading to the removal of 0.20% of records in Africa, 0.11% in the Americas, 0.11% in South-East Asia and the Western Pacific, and 0.87% in the Eastern Mediterranean – no more than 1.3% was removed for any one country. The final database included data on 1 263 119 individuals: 909 368 women and 353 751 men. Appendix B (available from: http://archive.lstmed.ac.uk/4566/) lists the data sources included in the final database. 

**Table 1:** Data used to derive weight-for-age reference curves for malaria-endemic countries in the WHO Regions of Africa, the Americas, South-East Asia and Western Pacific, 1995–2012

Data set and source	No. of countries	No. of studies	No. of individuals
**Initial database**			
Demographic and Health Surveys	56	111	1 266 209
Multiple Indicator Cluster Surveys	45	45	246 596
Household surveys using the WHO STEPwise approach	10	10	29 315
Other	26	95	733 201
**Database after cleaning^a^**			
Demographic and Health Surveys	53	106	870 430
Multiple Indicator Cluster Surveys	41	41	234 188
Household surveys using the WHO STEPwise approach	9	9	23 802
Other	25	48	203 516
**Final database^b^**			
Africa	38	140	681 022
Americas	13	29	302 279
South-East Asia and the Western Pacific	13	23	279 818
Total	64	192	1 263 119

WHO: World Health Organization.

^a^ Cleaning involved excluding: data with missing or invalid entries for country, sex, body weight or age; duplicate data; treatment data; data not representative of the population; and data from areas where malaria was not endemic.

^b^ For the final data set, only individuals older than 14 days and younger than 50 years were included and individuals with improbable weight-for-age *Z* scores were excluded. In addition, data from the WHO Eastern Mediterranean Region were excluded.

**Fig. 1:**
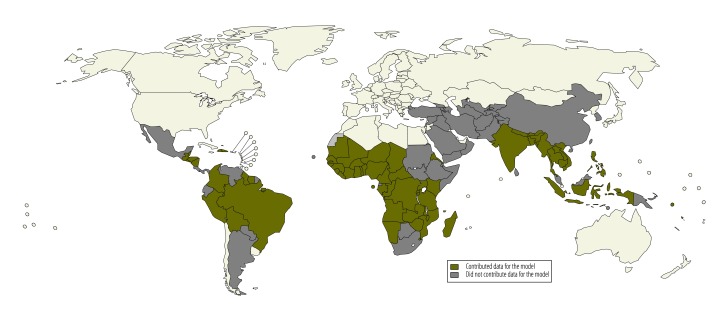
Malaria-endemic countries, 2012

As shown in Fig. 1, our data covered 64 of the 102 (62.7%) malaria-endemic countries for which malaria risk data were available. More than 80% of the data came from standardized health surveys, whereas the remaining data were obtained through literature and web searches or from public or private institutions or individual researchers. Sources included demographic surveillance systems, school surveys, health screening records, observational cohort studies and randomized controlled trials.

Data source: Malaria Atlas Project.^17^

**Fig. 2:**
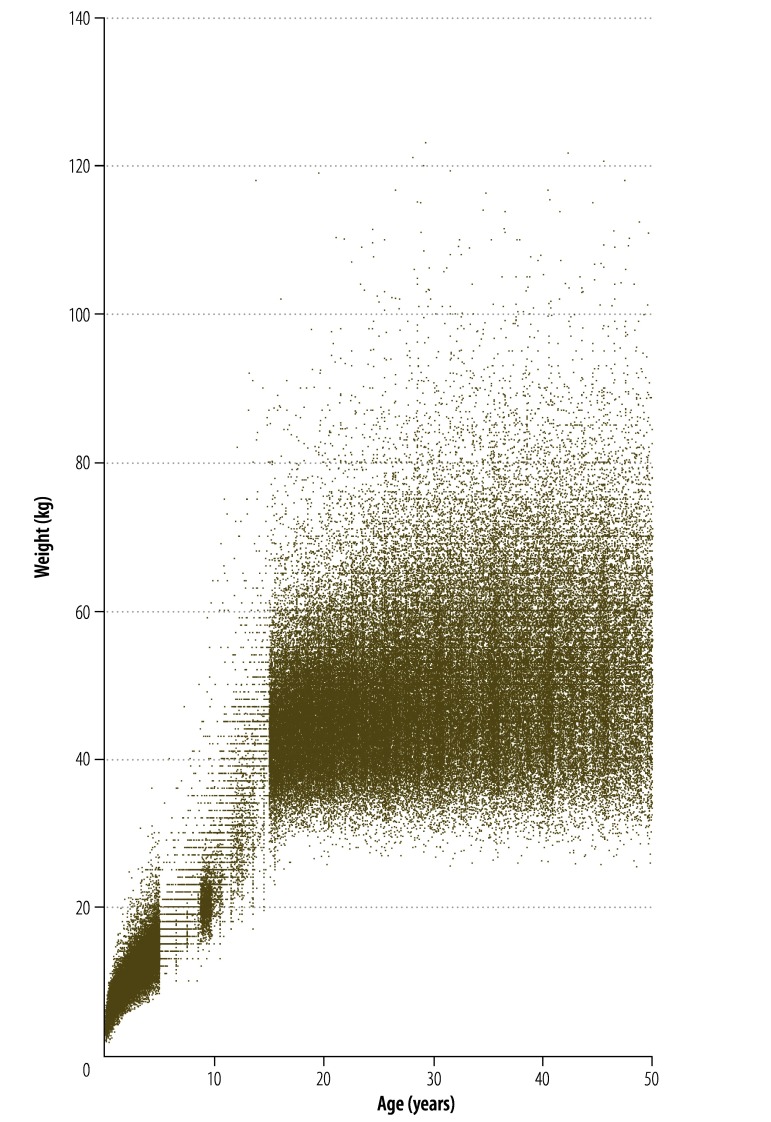
Weight-for-age scatter plot for females in malaria-endemic countries in the WHO South-East Asia and Western Pacific Regions 1995–2012

**Fig. 3:**
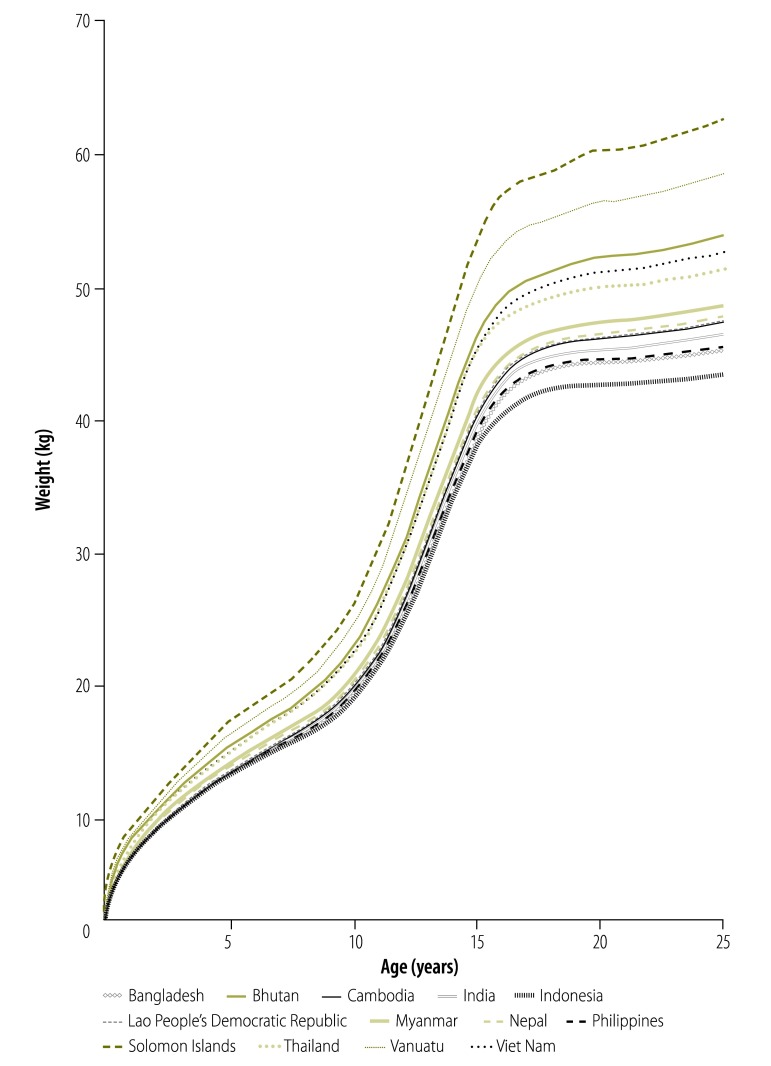
Median weight-for-age curves for females in malaria-endemic countries in the WHO South-East Asia and Western Pacific Regions, by country, 1995–2012

The availability of data varied substantially by age and sex between countries. Overall, 44.2% (558 218 individuals) of the data were for children younger than 5 years and 47.3% (596 883 individuals) were for women of childbearing age (i.e. aged 15–49 years). Only 2.5% (31 021 individuals) were for adult males (i.e. aged 18 years or older) and 3.8% (47 581 individuals) were for school-aged children (i.e. aged 6–12 years). Several non-DHS-type data sets showed signs of rounding and heaping of weights and ages. Data covering the full age range for both sexes were available for eight countries in Africa, two in South-East Asia and the Western Pacific, and one in the Americas. Fig. 2 shows an example of a weight-for-age scatter plot (plots for other regions and both sexes are in Appendix C, available from: http://archive.lstmed.ac.uk/4566/) and Fig. 3 shows a smoothed, country-level curve of weight-for-age medians (curves for other regions and both sexes are in Appendix D, available from: http://archive.lstmed.ac.uk/4566/).

WHO: World Health Organization.

WHO: World Health Organization.

**Fig. 4:**
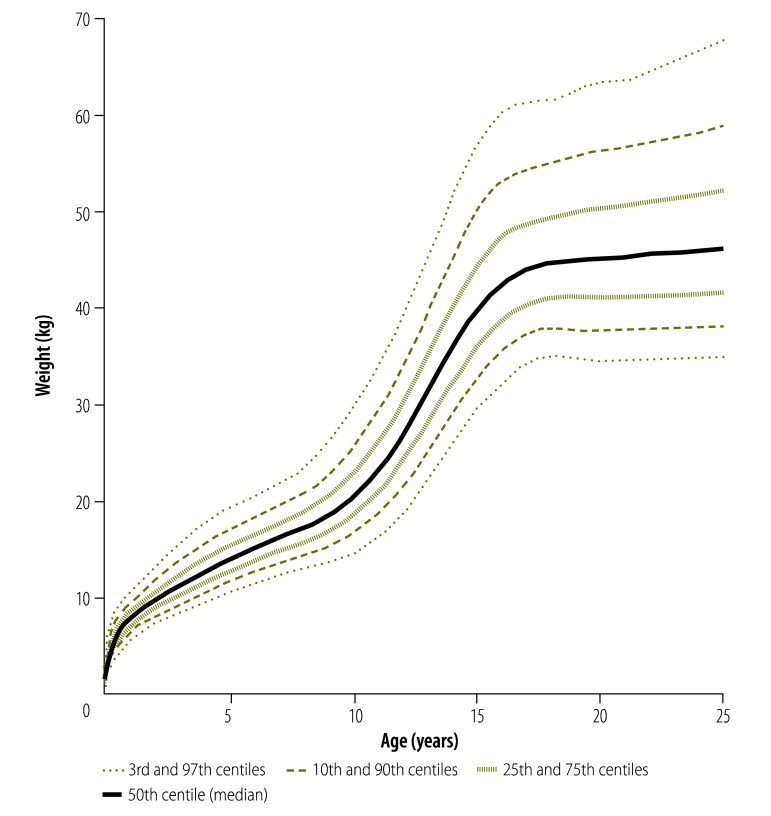
Weight-for-age reference curves for females in malaria-endemic countries in the WHO South-East Asia and Western Pacific Regions by selected centiles, 1995–2012

Appendix A lists the number of degrees of freedom that provided the best model fit for the reference curves in each region as derived using the Box–Cox power exponential model with cubic or penalized splines. Smoothed, regional, weight-for-age references were obtained for both sexes by combining country-level distributions weighted for the size of the population in malaria-endemic areas. Fig. 4 shows weight-for-age reference curves for different centiles for females in South-East Asia and the Western Pacific (curves for other regions and both sexes are in Appendix E, available from: http://archive.lstmed.ac.uk/4566/). Comprehensive tables containing Box–Cox power exponential model estimates, weight-for-age *Z* scores and all the centiles needed to reproduce the regional references can be downloaded from the WorldWide Antimalarial Resistance Network.^18^

WHO: World Health Organization.

### Model validation

Country-level, weight-for-age references were validated using data from the initial data set reserved for validation. Appendix F (available from: http://archive.lstmed.ac.uk/4566/) shows the percentage of individuals in the validation set that fell below our calculated 3rd, 10th, 50th, 90th and 97th centiles for both sexes in each individual country in the three regions. Most modelled centiles were well on target: 96.9% of centiles in the validation set fell within 3 percentage points of the modelled weight-for-age. Of the centile points tested, 2.0% lay between 3 and 5 percentage points from the modelled weight-for-age and 1.1% lay more than 5 percentage points distant. Deviations beyond 3 percentage points were more frequent in countries for which data were relatively scarce, such as Belize, the Philippines, the Solomon Islands and Suriname.

### Comparison between references

**Fig. 5:**
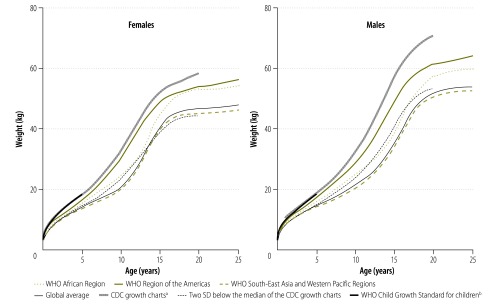
Model and standard weight-for-age curves in malaria-endemic countries, 1995–2012

CDC: Centers for Disease Control and Prevention; SD: standard deviation; WHO: World Health Organization.

^a^ Centers for Disease Control and Prevention growth charts for 2000.^15^

^b^ WHO Child Growth Standards for children aged 0–59 months.^5^

**Fig. 6:**
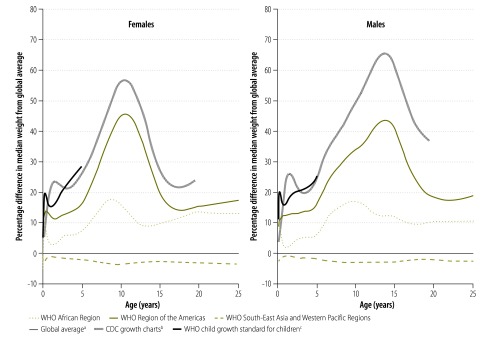
Difference in median weight-for-age values in malaria-endemic countries between the modelled global reference curve and modelled regional curves and standard curves, 1995–2012

CDC: Centers for Disease Control and Prevention; WHO: World Health Organization.

^a^ A global average was obtained by combining the three regional weight-for-age reference curves, weighted for the population at risk of malaria in each region.

^b^ Centers for Disease Control and Prevention growth charts for 2000.^15^

^c^ WHO Child Growth Standards for children aged 0–59 months.^5^

Modelled weight-for-age distributions differed considerably between the three regions (Fig. 5). Overall, body weight in South-East Asia and the Western Pacific was lower than in Africa or in the Americas. The median weight of an adult male aged 18 years or older in Africa and the Americas was 64.2 kg and 68.1 kg, respectively, whereas the median was 62.1 kg in South-East Asia and the Western Pacific. The corresponding median weights in females were 57.4 kg in Africa, 60.4 kg in the Americas and 50.0 kg in South-East Asia and the Western Pacific. Fig. 6 shows the percentage difference between median weight-for-age values in different regions and corresponding median values in our modelled global reference. Differences were most marked during the onset of the growth spurt in early adolescence. The modelled global reference curves are similar to curves for South-East Asia and the Western Pacific because the population at risk of malaria in this region was large.

Fig. 5 and Fig. 6 also show that median weight-for-age values in the three regions are much lower for all ages than in recent CDC growth charts – Fig. 5 shows that they are, at times, similar to values 2 SD below the medians in these charts. The difference in median weight between our modelled global reference and the growth chart median was around 20% for 1-year-olds and almost 70% for adolescents aged 13–14 years (Fig. 6). The peak difference in median weight between continents occurred during adolescence, which reflects the late onset of puberty in South-East Asia and the Western Pacific relative to Africa and America.

## Discussion

We developed regional weight-for-age growth references representative of populations in malaria-endemic areas of Africa, the Americas and South-East Asia and the Western Pacific using existing anthropometric data. To our knowledge, these are the first region-specific estimates of the weight achieved by age in these populations. Although our reference values were tailored to optimize antimalarial regimens, they are relevant to age-based therapies used for other diseases. Our modelling method was specifically designed such that data from a wide range of sources could be pooled to create robust, regionally representative references. The results demonstrate that large differences in weight-for-age distributions existed between the three regions and between these regions and optimal growth curves developed by WHO, which indicates that age-based dosing should shift from global to region-based regimens.

In all three regions, our models achieved good fits with the original data, which confirms that our generalized additive model for location, scale and shape extension method is a robust way of establishing growth references using mixed-source data in situations where multicentre growth reference studies are not feasible. The relative homogeneity of countries within the regions justifies modelling pooled country data sets in each region. Validation using independent data showed that the fitted country-level references adequately modelled the empirical distributions in each country.

The large majority of our data came from DHS, which are representative of national or subnational populations and have well-established designs and quality assurance methods. Extensive data were available for most of the age range of interest (i.e. 0–5 years and 15–49 years), which helped in modelling the age range for which fewer data were available (i.e. 5–14 years). Data for adolescents and adult males were missing for many countries, however, they were available across the full age spectrum for at least one country in each region. Since our two-step modelling approach was designed to use data from adjacent countries and age groups, where these showed similar growth distributions, we were able to model the growth curves for adolescents and adult males for all three regions. In the Eastern Mediterranean no data were available for older children or adult males. Several non-DHS-type data sets showed heaping that resulted from rounding of crude weight and age values. As heaped entries made up a very small proportion of the observations, however, the effect was limited and did not influence the final models.

To increase the representativeness of our curves, we did not use data sets from before 1995: 167 of 192 (87%) sets were from surveys conducted in 2000 or later. Although obesity is becoming more common in several low- and middle-income countries, only a wealthier subpopulation living in urban areas is likely to be affected, not the population in the rural areas most at risk of malaria. However, growth curves should be updated periodically (e.g. every 5 to 10 years) using the latest data from representative surveys. Our multisource modelling method provides a dynamic framework that enables new data to be incorporated when they become available and makes it possible to include non-malaria-endemic countries in the three regions modelled. The growth charts we generated for individual countries as part of the modelling process could serve as powerful public health tools to support decision-making at a national level. The logical next step would be to further validate these charts for countries where data were limited. Country-specific growth references could be improved further by extrapolating female data to derive male growth curves.

Our method provides a way of deriving regional growth references by collating weight-for-age data available for populations. Furthermore, our method facilitates the transition from generic, universal, age-based dosing practices to more data-driven, optimized, regional regimens for antimalarials. The method could also help monitor nutrition and optimize age-based dosing of other drugs.

## References

[1] Terlouw DJ, Courval JM, Kolczak MS, Rosenberg OS, Oloo AJ, Kager PA, et al. Treatment history and treatment dose are important determinants of sulfadoxine-pyrimethamine efficacy in children with uncomplicated malaria in Western Kenya. J Infect Dis. 2003 2 1;187(3):467–76. 10.1086/367705 12552431

[2] Terlouw DJ, Nahlen BL, Courval JM, Kariuki SK, Rosenberg OS, Oloo AJ, et al. Sulfadoxine-pyrimethamine in treatment of malaria in Western Kenya: increasing resistance and underdosing. Antimicrob Agents Chemother. 2003 9;47(9):2929–32. 10.1128/AAC.47.9.2929-2932.2003 12936996 PMC182608

[3] Taylor W, Terlouw DJ, Olliaro PL, White NJ, Brasseur P, ter Kuile FO. Use of weight-for-age-data to optimize tablet strength and dosing regimens for a new fixed-dose artesunate-amodiaquine combination for treating falciparum malaria. Bull World Health Organ. 2006 12;84(12):956–64. 10.2471/BLT.06.031492 17242831 PMC2627569

[4] Guerin PJ; on behalf of the WWARN ASAQ dose impact study group. Treatment efficacy of artesunate-amodiaquine treatment regimens for uncomplicated falciparum malaria: comparison of fixed versus co-blister formulations. In: 61st Annual Meeting of the American Society of Tropical Medicine and Hygiene (ASTMH); 2012 Nov 11–15; Atlanta, USA. Deerfield: American Society of Tropical Medicine and Hygiene; 2012.

[5] WHO child growth standards: methods and development. Length/height-for-age, weight-for-age, weight-for-length, weight-for-height and body mass index-for-age [Internet]. Geneva: World Health Organization; 2006. Available from: http://www.who.int/childgrowth/standards/technical_report/en/ [cited 2014 Oct 27].

[6] de Onis M, Garza C, Victora CG, Onyango AW, Frongillo EA, Martines J. The WHO Multicentre Growth Reference Study: planning, study design, and methodology. Food Nutr Bull. 2004 3;25(1) Suppl:S15–26. 15069916 10.1177/15648265040251S103

[7] de Onis M, Onyango AW, Borghi E, Siyam A, Nishida C, Siekmann J. Development of a WHO growth reference for school-aged children and adolescents. Bull World Health Organ. 2007 9;85(9):660–7. 10.2471/BLT.07.043497 18026621 PMC2636412

[8] Boerma JT, Sommerfelt AE. Demographic and health surveys (DHS): contributions and limitations. World Health Stat Q. 1993;46(4):222–6. 8017081

[9] The DHS Program Demographic and Health Surveys [Internet]. Calverton: United States Agency for International Development; 2012. Available from: http://dhsprogram.com[cited 2014 Nov 3].

[10] Cole TJ, Green PJ. Smoothing reference centile curves: the LMS method and penalized likelihood. Stat Med. 1992 7;11(10):1305–19. 10.1002/sim.4780111005 1518992

[11] Stasinopoulos DM, Rigby RA. Generalized additive models for location scale and shape (GAMLSS) in R. J Stat Softw. 2007;23(7)

[12] van Buuren S, Hayes DJ, Stasinopoulos DM, Rigby RA, ter Kuile FO, Terlouw DJ. Estimating regional centile curves from mixed data sources and countries. Stat Med. 2009 10 15;28(23):2891–911. 10.1002/sim.3667 19691045

[13] World malaria report 2011. Geneva: World Health Organization; 2011. Available from: http://www.who.int/malaria/world_malaria_report_2011/en/ [cited 2014 Nov 3].

[14] Hay SI, Okiro EA, Gething PW, Patil AP, Tatem AJ, Guerra CA, et al. Estimating the global clinical burden of *Plasmodium falciparum* malaria in 2007. PLoS Med. 2010 6;7(6):e1000290. 10.1371/journal.pmed.1000290 20563310 PMC2885984

[15] Kuczmarski RJ, Ogden CL, Grummer-Strawn LM, Flegal KM, Guo SS, Wei R, et al. CDC growth charts: United States. Adv Data. 2000 6 8; (314):1–27. 11183293

[16] van Buuren S, Fredriks M. Worm plot: a simple diagnostic device for modelling growth reference curves. Stat Med. 2001 4 30;20(8):1259–77. 10.1002/sim.746 11304741

[17] Population at risk. Oxford: Malaria Atlas Project; 2014. Available from: http://www.map.ox.ac.uk/explore/populations-risk/ [cited 2014 Nov 18].

[18] Age-based dose-regimen optimisation [Internet]. Oxford: WorldWide Antimalarial Resistance Network; 2014. Available from: http://www.wwarn.org/en/partnerships/projects/age-based-dose-regimen-optimisation [cited 2014 Nov 8].

